# The Canadian Heart Failure (CAN-HF) Registry: A Canadian Multicentre, Retrospective Study of Inpatients With Heart Failure

**DOI:** 10.1016/j.cjco.2022.04.005

**Published:** 2022-04-28

**Authors:** Stephanie Poon, Carlos Rojas-Fernandez, Sean Virani, George Honos, Robert McKelvie

**Affiliations:** a Sunnybrook Health Sciences Centre, University of Toronto, Toronto, Ontario, Canada; b Novartis Pharmaceuticals Canada Inc., Montreal, Quebec, Canada; c University of British Columbia, Vancouver, British Columbia, Canada; d Center Hospitalier de l'Université de Montréal, Montreal, Quebec, Canada; e St Joseph’s Health Care, Western University, London, Ontario, Canada

## Abstract

**Background:**

Despite recent advances in the management of patients with heart failure (HF), national data regarding the quality of care provided are lacking. The Canadian Heart Failure (CAN-HF) Registry was designed to obtain contemporary, real-world data describing the management of patients with HF.

**Methods:**

Quality of care in patients admitted for acute HF (AHF), in relation to guidelines and national HF quality indicators, was assessed as part of the CAN-HF Registry study.

**Results:**

A total of 943 patients admitted to the hospital with AHF were included in this analysis. Patient weight was not recorded on admission for 26% of patients, with daily weight being captured in only 61% of patients. Only 54% of inpatients received left ventricular ejection fraction assessment while hospitalized. Patient education was documented in 31% of patients prior to discharge, with 51% receiving instructions to follow up with a specialist upon discharge, and 2% being referred to a cardiac rehabilitation program. Although use of guideline-directed medical therapy increased during hospitalization, the proportions of patients receiving renin-angiotensin-aldosterone inhibition (63%), beta-blockade (80%), and mineralocorticoid receptor antagonist (40%) upon discharge indicate that potential room for improvement exists.

**Conclusions:**

The CAN-HF Registry study demonstrated a potential quality-of-care gap in the management of patients admitted with AHF.

## Résumé

**Contexte:**

Malgré les progrès récents dans la prise en charge des patients souffrant d’insuffisance cardiaque (IC), on note un manque flagrant de données nationales sur la qualité des soins prodigués. Le Registre canadien sur l’insuffisance cardiaque (*Canadian Heart Failure Registry* ou CAN-HF) a été conçu pour colliger des données contemporaines du monde réel décrivant la prise en charge des patients atteints d’IC.

**Méthodologie:**

Dans le cadre de l’étude *CAN-HF Registry*, on a évalué la qualité des soins prodigués aux patients hospitalisés pour cause d’IC aiguë (ICA) par rapport aux lignes directrices et aux indicateurs nationaux de qualité applicables à l’IC.

**Résultats:**

Notre analyse a porté sur 943 patients hospitalisés pour cause d’ICA. Le poids corporel n’avait pas été noté chez 26 % des patients au moment de leur admission à l’hôpital. Les mesures quotidiennes du poids avaient été prises chez 61 % des patients seulement. L’évaluation de la fraction d’éjection ventriculaire gauche avait été effectuée chez seulement 54 % des patients hospitalisés. Selon les documents consultés, 31 % des patients avaient reçu de l’information et des instructions avant de recevoir leur congé de l’hôpital; 51 % avaient reçu la directive de consulter un spécialiste pour assurer leur suivi après leur sortie de l’hôpital et 2 % avaient été orientés vers un programme de réadaptation cardiaque. Si le traitement médical administré durant l’hospitalisation était davantage conforme aux lignes directrices, les proportions de patients ayant reçu des inhibiteurs de la rénine-angiotensine-aldostérone (63 %), des bêtabloquants (80 %) et des antagonistes des récepteurs minéralocorticoïdes (40 %) à leur sortie de l’hôpital indiquent qu’il y a encore des progrès à faire.

**Conclusions:**

L’étude *CAN-HF Registry* a démontré qu’il pouvait y avoir des lacunes dans la qualité des soins prodigués aux patients hospitalisés pour cause d’ICA.

Advances in medical and device therapies in the past decade have decreased hospitalizations and reduced cardiovascular mortality for patients with heart failure (HF).^1^ Despite these advances and the publication of numerous HF clinical practice guidelines, a recent study by the Canadian Cardiovascular Society (CCS) HF Quality Indicators (QIs) Working Group and the Canadian Institute for Health Information demonstrated that 30-day readmission rates for patients with HF have remained unchanged, at approximately 20%, from 2009 to 2018,^2^ highlighting the need for further research to identify potential gaps in the care received by patients with HF.

Quality assurance is a process whereby healthcare organizations ensure that the care delivered to treat an illness meets accepted standards.^3^ Use of clinical practice guidelines is one component of the strategy to improve healthcare. However, this must be combined with approaches that quantify the quality of healthcare provided to patients, so that care gaps are identified and closed, and to shed light on systemic inequities in health service delivery. To this end, 49 HF QIs were established by the CCS HF QIs Working Group to track adherence to evidence-based safety and process indicators and clinical outcomes of HF care.^4^ However, feasibility testing revealed that only 30-day readmission could be measured across Canada in the absence of a cohesive system for data capture.^4^

The **Can**adian **H**eart **F**ailure (CAN-HF) Registry was designed to provide a contemporary description of HF management across the continuum of care, from outpatient clinics to inpatient settings. The overarching goal of the CAN-HF Registry was to obtain comprehensive real-world Canadian data from patients who present to the hospital for inpatient management of an acute HF (AHF) event, and for patients referred to hospital-based outpatient clinics for chronic HF management. The CAN-HF Registry provides a unique opportunity to comprehensively assess the quality of HF care that encompasses the entire patient journey. The objective of this report was to identify gaps in the quality of care of patients admitted with AHF.

## Methods

### Design

CAN-HF was a retrospective, observational, nonrandomized study of patients with HF. The study cohort consisted of patients aged 17 to 95 years who were either (i) inpatients whose primary reason for admission was AHF, or (ii) ambulatory patients treated for chronic HF in 7 sites across Quebec, Ontario, Manitoba, and British Columbia between January 2017 and April 2020. Sites were located in urban areas, and 5 of the 7 sites were teaching centres with access to HF specialists; the other 2 sites were community-based hospitals that did not have dedicated cardiology wards. The scope of the current analysis is limited to patients hospitalized with AHF; outpatient data will be analyzed separately in a subsequent publication. No exclusion criteria were applied. Clinical data were abstracted from the medical record into a secure, Web-based, electronic data-capture platform designed by IQVIA (Kirkland, Quebec, Canada). Baseline patient data reported by healthcare providers included patient demographics, HF history, comorbidities, vital signs, laboratory values, left ventricular ejection fraction (LVEF), and concurrent cardiovascular medication doses. Information regarding patient education, discharge disposition, and follow-up appointments was obtained at the point of discharge for inpatients. All information was captured based on available documentation in the patient’s medical record, including HF diagnosis. Study-site research personnel received training from IQVIA in the use of the electronic data-capture platform prior to the start of data collection and entry. Participating study sites obtained ethics review board approval prior to study commencement.

### Sample size and statistical analysis

Given the descriptive study design, no formal sample-size justification was performed. Based on the monthly average number of patients seen, we aimed to identify patients in an expedient manner within a brief timeframe (3 to 6 months). Each site was expected to identify a total of 100 inpatients for an intended sample size of 700 patients. Unless otherwise specified, data are reported as mean ± standard deviation (SD).

## Results

### Patient characteristics

The present analysis included 943 patients admitted to the hospital with AHF. A total of 793 patients (84%) were admitted from the emergency department (ED), and 150 (16%) were admitted directly to the ward. Baseline characteristics are reported in Table 1. In this study cohort, 490 patients (52%) had a diagnosis of HF with reduced ejection fraction (HFrEF), and 380 patients (40%) had a diagnosis of HF with preserved ejection fraction. The diagnosis was unknown for 73 patients (8%). The mean (SD) age for the combined sample was 76 (±14) years; 63% were age ≥75 years; and 56% were male. The majority of patients (58%) had not been admitted to the hospital for HF in the previous 12 months, and 31% did not have a prior history of HF. The most common comorbidities were hypertension (68%), coronary artery disease (43%), atrial fibrillation (43%), and diabetes (40%). The presumed etiologies of the underlying cardiomyopathy were ischemic heart disease (38%), nonischemic cardiomyopathy (19%), valvular heart disease (16%), and hypertensive heart disease (13%). The most commonly cited causes for HF decompensation were unknown (33%), infection (17%), arrhythmia (15%), ischemia (14%), nonadherence to fluid and sodium restriction (11%), and nonadherence to HF medication instructions (8%). Patients with HF with preserved ejection fraction tended to be older, female, and have higher rates of comorbidities, including hypertension and atrial fibrillation, but lower rates of coronary artery disease, in comparison to patients with HFrEF.

**Table 1:** Baseline characteristics

Characteristic	Combined sample (N)	HFrEF	HFpEF
	943	490 (52.0)	380 (40.3)
Age, y			
Mean (SD)	76 (14)	72(15)	80 (11)
Median (IQR)	80 (71–90)	75 (64–86)	83 (77–89)
< 55	82 (8.7)	68 (13.9)	13 (3.4)
55–64	98 (10.4)	67 (13.7)	27 (7.1)
65–74	171 (18.1)	107 (21.8)	55 (14.5)
≥ 75	592 (62.8)	248 (50.6)	285 (75.0)
Sex			
Male	531 (56.3)	336 (68.6)	164 (43.2)
Female	412 (43.7)	154 (31.4)	216 (56.8)
Comorbidities			
Hypertension	639 (67.8)	313 (63.9)	275 (72.4)
Coronary disease	408 (43.3)	236 (48.2)	148 (39.0)
Diabetes	375 (39.8)	186 (38.0)	164 (43.2)
Atrial fibrillation	408 (43.3)	189 (38.6)	186 (49.0)
Dyslipidemia	314 (33.3)	179 (36.5)	123 (32.4)
CKD	334 (35.4)	161 (32.9)	145 (38.2)
Cerebrovascular disease	154 (16.3)	72 (14.7)	67 (17.6)
Weight measured	698 (74.0)	367 (74.9)	290 (76.3)
Mean (SD), kg	82.2 (25.8)	83.1 (25.1)	81.2 (25.8)
Heart rate measured, bpm	914 (96.9)	471 (96.1)	371 (97.6)
Mean (SD), bpm	84.8 (20.4)	85.9 (20.4)	83.7 (20.3)
SBP measured	911 (96.6)	471 (96.1)	368 (96.8)
Mean (SD), mm Hg	132 (26)	126 (25)	139 (26)
DBP measured,	909 (96.4)	470 (95.9)	367 (96.6)
Mean (SD), mm Hg	73 (15)	73 (15)	72 (14)
LVEF measured	511 (54.2)	316 (64.5)	194 (51.1)
Mean (SD), %	39 (17)	28 (10)	56 (9)
Median (IQR), %	35 (20-50)	28 (20-35)	58 (53-62)
Min; max, %	7; 78	7; 60	15; 78
NYHA class measured	386 (40.9.0)	225 (46.0)	147 (38.7)
I	10 (2.5)	8 (3.6)	1 (0.7)
II	64 (16.2)	55 (24.4)	9 (6.1)
III	173 (43.7)	101 (44.9)	69 (46.9)
IV	139 (35.1)	56 (24.9)	68 (46.3)
HF history			
Yes	606 (64.3)	318 (64.9)	253 (66.6)
No	295 (31.3)	160 (32.7)	106 (27.9)
Unknown	42 (4.5)	12 (2.5)	21 (5.5)
Admitted to hospital for HF in the past 12 mo			
Yes	284 (30.1)	151 (30.8)	118 (31.1)
No	548 (58.1)	303 (61.8)	200 (52.6)
Unknown	111 (11.8)	36 (7.4)	62 (16.3)
Primary cause of the underlying cardiomyopathy			
Ischemic heart disease	360 (38.2)	237 (48.4)	104 (27.4)
Nonischemic cardiomyopathy	179 (19.0)	129 (26.3)	45 (11.8)
Valvular heart disease	146 (15.5)	68 (13.9)	72 (18.9)
Hypertensive heart disease	122 (12.9)	54 (11.0)	62 (16.3)
Other	94 (10.0)	32 (6.5)	54 (14.2)
Unknown	203 (21.5)	59 (12.0)	107 (28.2)
Length of hospital stay			
# of days (%)	869 (100.0)	454 (100.0)	354 (100.0)
Mean (SD)	10.4 (16.1)	10.4 (9.5)	11.00 (22.4)
Median (IQR)	7 (3-11)	8 (4-13)	7 (3-11)
Min; max	0; 375	0; 82	0; 375

Values are n (%), unless otherwise indicated.

bpm, beats per minute; CKD, chronic kidney disease; DBP, diastolic blood pressure; HF, heart failure; HFrEF, HF with reduced ejection fraction; HFpEF, HF with preserved ejection fraction; IQR, interquartile range; LVEF, left ventricular ejection fraction; max, maximum; min, minimum; NYHA, New York Heart Association; SBP, systolic blood pressure; SD, standard deviation.

Mean blood pressure and heart rate at the time of presentation were 132/73 (± 26/15) mm Hg and 85 (± 20) beats per minute, respectively (Table 1). The median (interquartile range [IQR]) systolic blood pressure was 129 (112-147) mm Hg. New York Heart Association (NYHA) class was documented in 42% of the patients; most patients had NYHA III (44%) or NYHA IV (35%) symptoms (Table 1). Mean LVEF was 39% (± 17%). Median LVEF was 35% (IQR: 20%-50%). Mean (SD) and median (IQR) length of hospital stay were 10 (± 16) and 7 (3-11) days, respectively (Table 1). Estimated glomerular filtration rate and natriuretic peptide data were captured for 38% and 27% of all patients, respectively (Table 2), and troponin (Tn) and high-sensitivity troponin data were captured for 24% and 47% of patients, respectively. Laboratory values were captured separately for patients admitted through the ED (Table 2) vs patients who were admitted directly to a hospital ward.

**Table 2:** Quality-improvement indicators

Indicator	Combined sample	HFrEF	HFpEF
Patients admitted	943 (100)	490 (52.0)	380 (40.3)
Laboratory values			
Serum sodium, mmol/L	838 (88.9)	417 (85.1)	353 (92.9)
	138.1 (± 5.0)	138.3 (± 5.0)	137.8 (± 5.3)
Serum potassium, mmol/L	837 (88.8)	416 (84.9)	352 (92.6)
	4.3 (± 0.8)	4.3 (± 0.8)	4.2 (± 0.7)
BUN, mmol/L	664 (70.4)	348 (71.0)	257 (67.6)
	13.7 (± 8.5)	13.4 (± 8.5)	14.3 (± 8.9)
Serum creatinine, μmol/L	829 (87.9)	409 (83.5)	354 (93.2)
	131.6 (± 72.3)	133.1 (± 76.5)	130.7 (± 69.1)
eGFR, mL/min per 1.73 m^2^	360 (38.2)	155 (31.6)	188 (49.5)
	50.4 (± 25.9)	51.8 (± 26.3)	49.5 (± 26.2)
Hemoglobin, g/L	820 (86.9)	402 (82.0)	349 (92.8)
	117.2 (± 22.8)	122.5 (± 23.6)	112.2 (± 21.5)
BNP, pg/mL	154 (16.3)	80 (16.3)	66 (19.5)
	1 505.7 (± 1672.1)	2061.7 (± 2 018.0)	843.0 (± 704.5)
NT- pro BNP, pg/mL	100 (10.6)	43 (8.8)	55 (14.5)
	9 894.4 (± 10,003.7)	13,374.0 (± 11,179.4)	7,261.5 (± 8, 265.8)
Troponin, ug/L	193 (24.3)	82 (16.7)	99 (26.1)
	0.2 (± 0.8)	0.2 (± 0.5)	0.2 (± 1.0)
hsTroponin, ng/L	376 (47.4)	231 (47.1)	152 (40.0)
	74.0 (±133.4)	74.6 (± 108.5)	71.9 (± 169.0)
Chest X-ray	772 (82.1)	371 (75.7)	331 (87.1)
ECG	791 (84.10)	386 (78.8)	340 (89.5)
Specialty consultation related to this HF episode for patients admitted through ED			
Yes	599 (75.50)	300 (77.9)	271 (79.9)
No	163 (20.6)	66 (17.1)	57 (16.8)
Specialty consulted			
Cardiology	379 (63.3)	223 (74.3)	152 (56.1)
Endocrinology	4 (0.7)	1 (0.3)	2 (0.7)
Geriatrics	11 (1.8)	3 (1.0)	8 (3.0)
Internal medicine	219 (36.6)	82 (27.3)	116 (42.8)
Nephrology	11 (1.8)	5 (1.7)	6 (2.2)
Oncology	2 (0.3)	N/A	2 (0.7)
Pulmonology	16 (2.7)	7 (2.3)	8 (3.0)
Surgery	1 (0.2)	1 (0.3)	N/A
Other	25 (4.2)	16 (5.3)	6 (2.2)
Discharged from hospital alive	869 (92.2)	454 (92.6)	354 (93.2)
Patient referred to an outpatient clinic/program			
No	507 (58.3)	203 (44.7)	248 (70.1)
Yes	362 (41.7)	251 (55.3)	106 (29.9)

Values are n (%) or mean (± standard deviation); the latter are for the 793 patients admitted to the hospital via the ED.

BNP, brain natriuretic peptide; BUN, blood urea nitrogen; ECG, electrocardiogram; ED, emergency department; eGFR, estimated glomerular filtration rate; HF, heart failure; NT, N-terminal.

Laboratory values were not reported for the majority of patients who were admitted directly to a hospital ward (N = 150). Available laboratory data for patients admitted directly to the ward were generally consistent with data reported in Table 2 (ie, estimated glomerular filtration rate, 49.8 ± 33.6 ml/min per 1.73 m^2^; Tn, 0.3 ± 0.4 ug/L; sodium, 137.6 ± 4.3; potassium, 4.1 ± 0.7 mmol/L; blood urea nitrogen, 13.3 ± 9.6 mmol/L; hemoglobin, 120.3 ± 24.5), although lower brain natriuretic peptide (1141.3 ± 675.9 pg/mL) and N-terminal pro-brain natriuretic peptide levels (8673.4 ± 6297.8 pg/mL), and high-sensitivity troponin values (55.1 ± 59.6 ng/L), and higher serum creatinine levels (182.8 ± 126.9 μmol/L) were observed, compared to those for patients who were first admitted to the ED.

### Adherence to AHF hospital-phase QIs

Table 2 summarizes adherence to selected CCS HF QIs pertaining to the hospital and discharge/transition phases. Weight was recorded for 74% of patients on admission, whereas blood pressure and heart rate were measured in 97%. Overall, 574 patients (61%) had daily weights measured at least 80% of the time during their hospitalization.

Serum creatinine level was measured for 88% of patients at some point during their hospitalization, and sodium and potassium levels were obtained for 89% of patients. Overall, 799 patients (85%) had daily assessment of renal function and electrolytes. Chest radiographs (CXRs) and electrocardiograms (ECGs) were performed for 82% and 84% of patients, respectively, during their hospitalization; CXRs and ECGs were performed on the first day of admission to the ED for 87% and 92% of patients, respectively. Only 54% of inpatients had an LVEF determination while hospitalized.

Specialists were consulted on 599 (76%) of the 793 patients admitted to the hospital through the ED. The cardiology and internal medicine departments were consulted for 63% and 37% of patients, respectively. Specialty consultation information was not captured for the 150 patients admitted directly to wards.

Education about HF and instructions regarding dietary plus lifestyle recommendations were provided to 31% and 26% of patients, respectively, before discharge. Patients were mostly given verbal instruction (25%), whereas 15% were given booklets containing the information, and 0.2% had counselling via a support group. Information regarding patient education on disease area and dietary and lifestyle education was missing in 69.2% and 73.8% of patient records, respectively.

### Adherence to guideline-directed medical therapies (GDMTs) for patients with HFrEF

Use of GDMTs for treatment of HFrEF increased from admission to discharge (Table 3). Beta-blocker use increased from 62% to 80%; mineralocorticoid receptor antagonist (MRA) use increased from 21% to 40%; angiotensin-converting enzyme inhibitor (ACEi) and angiotensin receptor blocker (ARB) use increased from 45% to 57%; angiotensin receptor-neprilysin inhibitor (ARNI) use increased from 3.5% to 6.2%; and use of any ACEi/ARB/ARNI increased from 48% to 63%.

**Table 3:** Heart failure therapy

Medication class	All patients (N = 943)	All patients (N = 869)	Patients with HFrEF (n = 490)	Patients with HFrEF (n = 454)	Patients with HFpEF (n = 380)	Patients with HFpEF (n = 354)
	Admission	Discharge	Admission	Discharge	Admission	Discharge
ACEI/ARB	408 (43.3)	416 (47.9)	220 (44.9)	258 (56.8)	158 (41.6)	135 (38.1)
ARNI	17 (1.8)	29 (3.3)	17 (3.5)	28 (6.2)	N/A	1 (0.3)
MRA	168 (17.8)	264 (30.4)	104 (21.2)	183 (40.3)	60 (15.8)	75 (21.2)
Beta-blockers	595 (63.1)	626 (72.0)	304 (62.0)	365 (80.4)	249 (65.5)	222 (62.7)
Hydralazine	78 (8.3)	84 (9.7)	31 (6.3)	41 (9.03)	44 (11.6)	41 (11.6)
Nitrate	166 (18.0)	163 (18.8)	75 (15.3)	78 (17.2)	80 (21.1)	75 (21.2)
Ivabradine	3 (0.3)	2 (0.2)	3 (0.6)	2 (0.4)	N/A	N/A
Diuretics	636 (67.4)	712 (81.9)	294 (60.0)	355 (78.2)	287 (75.5)	304 (85.9)

Values are n (%), indicating medication classes at admission and discharge.

ACEi, angiotensin-converting enzyme inhibitor; ARB, angiotensin receptor blocker; ARNI, angiotensin receptor-neprilysin inhibitor; HFpEF, heart failure with preserved ejection fraction; HFrEF, heart failure with reduced ejection fraction; MRA, mineralocorticoid receptor antagonist; N/A, not applicable.

### Adherence to discharge/transition phase HF QIs

At discharge, 869 patients (92%) were alive. Within this cohort, the majority (83%) were discharged home, whereas 11% were transferred to a post-acute care rehabilitation unit or long-term care facility, with the remaining 6% captured as other or unknown. Overall, 443 of the discharged patients (51%) received instructions to follow up with a specialist, and 165 of the patients in this group were told to obtain appointments with their current cardiologists. A total of 321 patients (37%) were instructed to follow up with their family physicians. A total of 362 patients (42%) were referred to an outpatient clinic/program. Of these patients, 262 were referred to a heart function program, and 16 were referred to a cardiac rehabilitation program.

## Discussion

Data from this contemporary multicentre Canadian registry reveal significant gaps in the quality of care received by patients admitted with AHF, as defined by the CCS HF QI Project. Although most inpatients received an ECG and CXR on the day of admission, and daily assessment of electrolytes and renal function, only 61% had daily weights recorded more than 80% of time. Additionally, only 54% of patients had an assessment of LVEF during their hospitalization. Our data also revealed that a minority of patients (31%) received HF education before discharge, and that only 26% were given instructions regarding dietary or lifestyle recommendations. Although prescription of GDMT improved over the course of hospitalization, underutilization persists. Finally, approximately half of all patients admitted with AHF were not given specific instructions regarding follow-up appointments with specialists, and only 42% of discharged patients were referred to an outpatient clinic/program. Of note, almost all HF inpatients (98%) did not receive a referral to cardiac rehabilitation.

To our knowledge, this is the most comprehensive and contemporary analysis of adherence to national HF QIs in Canada. A limited feasibility assessment completed by the CCS HF QIs Working Group in 2015 revealed that only 30-day hospital readmission rate could be measured nationally.^4^ The CAN-HF Registry provides a unique opportunity to obtain more granular data on key safety and process QIs pertaining to care of patients admitted to the hospital with AHF.

Safety and process indicators captured by the CAN-HF Registry included daily assessments of blood chemistry panels and completion of a CXR and an ECG as part of the initial evaluation. As HF and its treatment can result in electrolyte and renal function abnormalities, volume status and perfusion must be monitored on a regular basis by daily assessment of body weight, blood pressure, serum electrolytes, and renal function.^5^^,^^6^ A CXR can assist in the diagnosis of AHF and should be obtained within 2-8 hours of initial assessment.^4^ Patients admitted to the hospital should have an ECG as part of their initial evaluation, as this is fundamental to identify causes of acute dyspnea and/or HF decompensation, which may require immediate action. Our analyses revealed that most inpatients received daily assessments of renal function and electrolytes, and a CXR and ECG on the same day as admission. Although blood pressure and heart rate were measured in most patients, only 61% had daily weights measured at least 80% of the time during their hospitalization. This finding highlights an important care gap, as daily weights are essential in guiding diuretic titration during a hospitalization and play a critical role in educating patients on self-care management strategies upon discharge.

Other process indicators captured by the CAN-HF Registry included assessment of LVEF, patient education, and use of GDMTs (particularly for patients with HFrEF). Patients with a documented history or working diagnosis of HF admitted to the hospital for HF should have an LVEF assessment if one has not been obtained in the prior 12 months. Knowledge of LVEF is fundamental for decisions on diagnosis, prognosis, therapy, and referral.^4^ However, only 54% of patients in the CAN-HF cohort had LVEF assessed during their hospitalization. The reasons for this are unclear; a contributing factor may be lack of timely access to echocardiography. An alternative possibility is that echocardiograms were performed without reporting of LVEF or were arranged early post-discharge for some patients. In any case, the fact that LVEF was documented for only half of the CAN-HF Registry inpatients highlights a common challenge in capturing this process indicator, which is critical in determining GDMT prescription adherence for patients with HFrEF.

As most patients (73%) in the CAN-HF Registry were admitted in 2019, prescription of GDMT must be evaluated in the context of CCS HF guidelines from that period. The 2017 CCS HF guidelines recommend that most patients with HFrEF be treated with “triple therapy,” which includes an ACEi (or ARB for ACEi-intolerant patients), a beta-blocker, and an MRA in the absence of contraindications.^6^ Our data indicated that 48% of patients were receiving an ACEi and/or ARB and/or ARNI at baseline, whereas 22% were receiving hydralazine (HDZ) and/or isosorbide dinitrate (ISDN) at the time of hospital admission. Considering a typical rate of ACEi intolerance of approximately 10%-20%,^6^ the fact that 22% of patients received HDZ or ISDN is unsurprising. Yet when considering ACEi and/or ARB and/or ARNI use in conjunction with HDZ and/or ISDN as a pillar of HFrEF GDMT, 30% of patients were not receiving this GDMT component at admission. Similarly, the proportions of patients receiving beta-blockers and MRAs indicate potential room for improvement, with 38% and 79% of patients with HFrEF, respectively, not receiving these therapies on admission. Recent analyses in ambulatory patients have suggested that up to a third of patients are eligible for but undertreated with inhibitors of the renin-angiotensin-aldosterone system and beta-blockers (BBs), and 19%-66% of patients are eligible for but undertreated with MRAs.^7^^,^^8^ Thus, the treatment gaps noted in the CAN-HF Registry are unlikely to be explained by treatment ineligibility or contraindications alone. What remains unclear is whether data from a specialized, multidisciplinary HF clinic at a quaternary academic centre^8^ are representative of treatment patterns more broadly, or how generalizable these data may be to the inpatient setting.

Although an important increase in GDMT use was documented by use of ACEis, and/or ARBs, and/or ARNIs (from 48% to 63%), beta-blockers (from 62% to 80%), and MRAs (from 20% to 40%) by the time of discharge, an opportunity to improve the care and outcomes of patients with HFrEF remains evident. The low absolute rate (3.5% at admission to 6.2% at discharge) of sacubitril or valsartan use is noteworthy, as 73% of the data were from 2019, and in 2014, the **P**rospective Comparison of **AR**Ni With **A**CEi to **D**etermine **I**mpact on **G**lobal **M**ortality and Morbidity in **H**eart **F**ailure (PARADIGM-HF) study clearly established the increased effectiveness of using sacubitril or valsartan rather than enalapril in reducing the risks of death and HF hospitalization in patients with HFrEF.^9^ Additionally, a recent study suggested that optimizing the use of sacubitril and valsartan in Canada could lead to over $40 million in savings related to hospitalizations alone.^10^ Likewise, our data reveal surprisingly low rates of ivabradine usage (< 1% at admission and discharge), given the observed mean heart rate of 85 beats per minute, suggesting broad eligibility for ivabradine use in this population. Gaps in adherence to GDMTs that exist in specialized HF care settings potentially can be explained by the limits of physiological factors rather than by clinical inertia; other nonmedical factors include medication costs, lack of reimbursement by payors, the expected lag time in uptake of new therapies, and limited access to healthcare facilities or specialists.^7^^,^^11^

Comparisons of the present analysis to other recent chart audit–based analyses of GDMT in patients with HFrEF have important limitations, given that the present analysis is focused exclusively on the inpatient setting, whereas the most recent comparable analyses have been limited to ambulatory patients. Two recent analyses from a similar timeframe in ambulatory patients documented overall higher rates of GDMT adherence than those observed in the present inpatient analysis. **Cha**nge the **M**anagement of **P**atients With **H**eart **F**ailure (CHAMP-HF) , a registry study of 3518 patients with chronic HFrEF in the US, documented use of ACEi and/or ARB and/or ARNI at 69%, use of BBs at 67%, and use of an MRA at 33% in eligible patients.^8^ Jarjour and colleagues also evaluated GDMT adherence from a retrospective chart review of 511 ambulatory patients seen in 2017 at an academic hospital-based multidisciplinary HF clinic, and they documented high rates of prescription in eligible patients across all classes of GDMT (ACEi or ARB: 83%, BB: 99%, MRA: 93%, ARNI: 91%).^7^ Limited contemporary analyses are restricted to inpatients. However, the **G**et **W**ith **t**he **G**uidelines (GWTG) registry study of 501,238 inpatients in the US (2010-2016) documented comparable rates of GDMT usage on admission, as well as a similar increase in GDMT usage between admission and discharge, as was documented in the present analysis.^12^ Patient education is another key process indicator of quality HF care, because HF requires patients (and/or their caregivers) to be actively involved in their care to prevent recurrent episodes of fluid retention. Essential education includes teaching patients how to recognize symptoms of worsening HF, adjust their diuretics according to their daily weights, and limit their sodium and/or fluid intake to maintain clinical stability.^3^^,^^4^^,^^13^ Despite the importance of patient education as a key process indicator of quality HF care, only 31% of patients were documented to have received disease and treatment education before discharge. However, information about patient education was missing or unknown for almost 70% of patients. Moreover, patient education did not appear to be completed in a standardized fashion, as 25% were given verbal instruction, 15% were provided with a booklet, and 0.2% had counselling via a support group. Our analysis suggests that there is room for improvement in ensuring and documenting that patients are given a comprehensive care plan upon discharge; the care plan ideally should be customized and should contain lifestyle management advice, as well as an algorithm to adjust diuretics according to daily weights.

Finally, important HF QIs at the point of discharge include early outpatient assessment for patients and referral to a cardiac rehabilitation program. Follow-up within 2 weeks of a HF hospitalization appears to be associated with a lower risk of recidivism at 30 days.^6^^,^^14^^,^^15^ Moreover, participation in a cardiac rehabilitation program (that includes regular exercise) is associated with improved outcomes, such as reduced mortality, lower risk of readmission, and higher quality of life; thus, referral to cardiac rehabilitation programs should be considered for all stable NYHA I to III HF patients.^16^^,^^17^ However, our analysis demonstrated that half of all inpatients in the CAN-HF Registry did not have documented instructions regarding follow-up appointments with specialists, and only 42% of discharged patients were referred to an outpatient clinic/program. Additionally, the vast majority of HF inpatients (98%) did not receive a referral to a cardiac rehabilitation program. Patients living in rural areas may not have access to a cardiac rehabilitation program, but most sites in the CAN-HF Registry are urban centres. Overall, our analysis reveals that there is ample opportunity to improve the quality of care that patients with HF are provided at the point of discharge, which will assist in ensuring a safe transition to the outpatient setting and preventing readmissions.

### Study limitations

Data from the CAN-HF Registry reflect the experience of patients from sites that elected to participate in the registry; thus, they may not be generalizable to all care practices. We did not capture data on past intolerances or specific contraindications to HF medications, nor was degree of frailty considered, both of which may correlate with medication use. Additionally, as CAN-HF data are based on documentation found within the medical record, the comprehensiveness of the data is limited by the specific information captured at each site.

## Conclusion

In this study of a contemporary registry of patients with HF in Canada, we have found that potential gaps remain in the quality of care provided to patients admitted with acute HF. Despite the availability of guidelines and publication of national HF QIs, documentation of LVEF, daily weights, patient education, follow-up appointments with specialists, use of GDMT, and referral to cardiac rehabilitation programs remains suboptimal. The CAN-HF Registry provided us with a unique opportunity to determine adherence to key safety and process Qis in the care of patients with HF. This study represents an important first step in the journey, which we hope will lead ultimately to future collaborative and sustained efforts, with the goal of improving the quality of care for our patients with HF in the decades to come.
